# Predictors of ischemic events in patients with unilateral extracranial vertebral artery dissection: A single-center exploratory study

**DOI:** 10.3389/fneur.2022.939001

**Published:** 2022-07-28

**Authors:** Yanhong Yan, Ziwei Lu, Yafang Ding, Jianhong Pu, Chunhong Hu, Zhongzhao Teng, Pinjing Hui

**Affiliations:** ^1^Department of Stroke Center, The First Affiliated Hospital of Soochow University, Suzhou, China; ^2^Department of Radiology, The First Affiliated Hospital of Soochow University, Suzhou, China; ^3^Department of Radiology, University of Cambridge, Cambridge, United Kingdom

**Keywords:** vertebral artery dissection, color duplex ultrasonography, high resolution magnetic resonance imaging, intraluminal thrombus, stroke

## Abstract

**Objective:**

Extracranial vertebral artery dissection (EVAD) is one of the main causes of stroke in young and middle-aged patients. However, the diagnosis is challenging. This study aimed to identify the characteristics of EVAD on color duplex ultrasonography (CDU) and high-resolution magnetic resonance imaging (hrMRI), hoping to improve the accuracy and determine the relative contribution of vessel findings and clinical factors to acute ischemic events.

**Methods:**

Patients with unilateral EVAD were recruited and divided into ischemia and non-ischemia groups. Clinical features of patients and the lesion location; a variety of signs which indicate dissection, including the presence of an intimal flap, double lumen, intramural hematoma, dissecting aneurysm, intraluminal thrombus, and irregular lumen; and other quantitative parameters of each dissected segment on CDU and hrMRI were reviewed, respectively. Multiple logistic regression was performed to explore the association between clinical, imaging characteristics, and ischemic events in patients with unilateral EVAD.

**Results:**

Ninety-six patients with unilateral EVAD who met the inclusion criteria were enrolled during a six-year period. Overall, 41 cases (42.7%) were confirmed as ischemic stroke (*n* = 40) or transient ischemic attack (*n* = 1) during the 48 h after the onset of symptoms. Men, infections during the last week, and smoking were more common in the ischemia group. Intraluminal thrombus and occlusion on CDU were more prevalent in patients with cerebral ischemia than in those without (36.6 vs. 5.5%; *p* < 0.001, and 39.0 vs. 9.1%; *p* = 0.001, respectively). On hrMRI, intraluminal thrombus and occlusion were also more frequent in the ischemia group than in the non-ischemia group (34.1 vs. 5.5%; *p* < 0.001, and 34.1 vs. 9.1%; *p* = 0.003, respectively). In addition, lesion length on hrMRI was significantly longer for patients with ischemia (81.5 ± 41.7 vs. 64.7 ± 30.8 mm; *p* = 0.025). In multivariable logistic regression analysis, male gender, infections during the last week, and the presence of intraluminal thrombus on CDU and hrMRI were independently associated with acute ischemic events.

**Conclusion:**

Male sex, infections during the last week, and the presence of intraluminal thrombus due to dissection are associated with an increased risk of ischemic events in patients with unilateral EVAD.

## Introduction

Extracranial vertebral artery dissection (EVAD) is increasingly recognized as an important cause of ischemic stroke in young and middle-aged patients, often caused by neck distortion, chiropractic manipulation, or blunt trauma (1–4). Patients with EVAD can have a wide range of symptoms, from completely asymptomatic to a severe stroke in the vertebro-basilary territory (5–7). Given its high risk of neurological deficit, an accurate and timely diagnosis is crucial (8, 9). It has been demonstrated that the clinical presentation of patients with EVAD depends on factors, including the lesion location, degree of obstruction, and functionality of collateral circulation (10–12). Invasive digital subtraction angiography (DSA) has been the gold standard for clinical decision-making during the past years (13). Recently, advanced high-resolution magnetic resonance imaging (hrMRI) offers excellent visualization of both the arterial wall and lumen, enabling the detection of multi-features of EVAD (10, 14, 15). However, both of them are time-consuming. Color duplex ultrasonography (CDU) is an easily accessible noninvasive imaging modality that can quickly delineate the lumen and arterial wall of the extracranial vertebral artery, as well as provide hemodynamic information (16, 17). However, the value of CDU for the diagnosis of EVAD and the prediction of ischemic events and treatment outcomes requires validation.

To the best of our knowledge, risk factors for cerebral ischemia in unilateral EVAD patients have not been systematically investigated. This study is designed to explore the characteristics of EVAD on CDU and validate CDU findings using hrMRI to determine high-risk clinical and imaging features associated with acute ischemic events.

## Materials and methods

### Study design and population

Between January 2016 and September 2021, patients with unilateral EVAD identified by CDU and later validated by hrMRI within 48 h after the onset of symptoms in our stroke center were consequently recruited. Our local ethics committee approved this study, and informed consent was obtained from all patients. CDU was performed on the day of admission. If EVAD was suspected, hrMRI would be performed in <24 h. The clinical characteristics were recorded, including sex, age, body mass index (BMI), history of trauma, smoking, alcohol use, hypertension (systolic blood pressure >140 or diastolic blood pressure >90), hyperlipidemia, diabetes mellitus, migraine (according to the International Headache Society criteria), blood test, and infections during the week preceding the onset of symptoms. Patients were classified into two groups according to clinical manifestations: (i) patients with local symptoms or signs only: headache, neck pain, or Horner syndrome; (ii) patients with ischemic events on the day of onset of symptoms, including ischemic stroke (>24 h) and transient ischemic attack (TIA) (<24 h). The National Institutes of Health Stroke Scale (NIHSS) was used to assess stroke severity on admission. The ischemic stroke status was determined by having the focal onset of neurological symptoms and positive diffusion-weighted imaging (DWI) (18).

Extracranial vertebral artery dissection was considered traumatic if patients had a recent history of severe trauma that directly impacted the head and/or neck area, which was associated with facial, basal skull, or cervical spine fractures (4). EVAD was defined as spontaneous in the absence of severe trauma or with minor cervical trauma, including cervical manipulation therapy, heavy load lifting, and excessive neck movements during sports and recreational activities.

The diagnosis of pulmonary infections was based on a comprehensive evaluation, taking into account the presenting characteristics (fever, cough with purulent sputum, or difficulty breathing, etc.), positive laboratory results, and the lesions of lung infection detected by high-resolution computed tomography (CT) within 1 week preceding the onset of symptoms caused by EVAD. The diagnosis of urinary tract infections was also based on a thorough analysis, including the clinical features (symptoms of frequent urination, urinary pain, or dysuria, etc.), positive laboratory examination results, and the presence of a culture of uropathogenic bacteria from the urine within the last week preceding the vascular event. Positive laboratory results of the initial examination were clinical markers of inflammation, including elevated high-sensitivity C-reactive protein (>3 mg/L) and high leukocyte counts defined as leukocyte counts >9.5 × 10^9^/L, neutrophil counts >6.3 × 10^9^/L, or monocyte counts >0.6 × 10^9^/L.

Exclusion criteria: (1) cervical and cerebral vasculopathy (e.g., atherosclerotic stenosis≥50% or occlusion, moyamoya disease, vasculitis; (2) evidence of cardioembolism; (3) previous strokes or transient ischemic attacks; and (4) subjects with stents; (5) hemorrhagic stroke detected by brain CT on admission.

### CDU examination and assessment

Color duplex ultrasonography was performed on all patients using a color-coded duplex machine (CX50, Philips, Netherlands) equipped with a compound imaging 12-3 MHz linear-array transducer and a 5-1 MHz curved array transducer. Patients were imaged in a supine position with the neck slightly extended.

Two senior sonographers independently reviewed all anonymized CDU images blinded to patient clinical information and hrMRI. The following lesion characteristics were recorded or measured, (1) intimal flap; (2) intramural hematoma, wall thickening with or without lumen stenosis; (3) double lumen, dissecting membrane with true and false lumen; (4) intraluminal thrombus; (5) dissecting aneurysm, and indirect signs such as segmental or successive dilation in diameter, irregular lumen, and tapering or occlusion of the lumen (19–21). The thrombus forming directly at the V1 segment, adjacent to an intimal flap, without extending to the V2 segment was defined as an intraluminal thrombus *in situ*, whereas those located in the V2 and or V3 segment, at the distal part of the whole lesion, forming downstream from the intramural hematoma were defined as distal intraluminal thrombus. Luminal stenosis was calculated following the NASCET criterion (22) as mild if stenosis <50%, moderate if stenosis ϵ [50, 69%], severe if stenosis ϵ [70, 99%], and occlusion. The dissection site was classified according to its location (23): V1, between the origin and entry into the transverse foramen of the C6 vertebra; V2, the mid-cervical course between processes C6 to C2 vertebrae; and V3, extending from the C2 transverse foramen to the dura mater of the foramen magnum. Any discrepancies between the two reviewers were resolved by consensus.

### hrMRI examination and assessment

All patients underwent hrMRI with a 3.0 T scanner combined with a cervicocerebral coil (Ingenia, Philips Healthcare, Netherlands). The hrMRI protocol included: T_1_-weighted image (T1W), contrast-enhanced T1W (CE-T1W), fat-suppressed T_2_-weighted image (FS-T2W), 3D proton density-weighted isotropic acquisition (3D PDW), and 3D time of flight magnetic resonance angiography (TOF). Standard brain magnetic resonance imaging (MRI) was implemented to confirm the acute ischemic lesions, including T1W, T2W, and DWI. The specific scanning parameters are detailed in the Supplementary materials.

Two senior neuroradiologists blinded to the clinical information reviewed all anonymized images independently on a dedicated workstation (IntelliSpace Portal v6.0). The following information and measurement were recorded, (1) intimal flap; (2) intramural hematoma; (3) double lumen; (4) intraluminal thrombus; (5) aneurysmal dilatation, as well as indirect signs such as enlargement of the outer contour, irregular lumen, string or occlusion (24, 25). The lesion length was measured using a spine line tool. NASCET-defined luminal stenosis was calculated on maximum intensity projection of TOF (22), mild if stenosis <50%, moderate if stenosis ϵ [50, 69%], severe if stenosis ϵ [70, 99%], and occlusion. The presence and extent of an acute ischemic lesion in posterior circulation territory were evaluated using the DWI-based posterior circulation Alberta Stroke Program Early CT Score (pc-ASPECTS) system (26). The reviewers resolved any discrepancies by consensus.

### Treatment and prognosis

According to the European Stroke Organization (ESO) guidelines (27) and patients' clinical presentations and examinations, all patients received optimal clinical treatment as determined by physicians. Functional outcomes at 6 months were assessed with the modified Rankin Scale (mRS) during outpatient visits, and scores 0 to 2 were defined as “favorable” outcomes, and scores higher than 2 were defined as “unfavorable” outcomes.

### Statistical analysis

Statistical analysis was performed with SPSS 19.0 (IBM Corp., Armonk, NY, USA). Patients' demographic and clinical characteristics were expressed as mean±standard deviation (SD), median [interquartile range (IQR)], or percentage where appropriate. Frequencies and categorical data were compared with Chi-Square or Fisher's exact test where appropriate, and continuous variables were compared using a *t*-test or the Mann-Whitney U test between 2 groups. Variables were selected for multivariable analyses if *p* ≤ 0.1 in the univariate analysis. Multivariable logistic regression analysis with the method of forwarding selection was conducted to determine independent predictors for ischemic events and treatment outcomes, respectively. We expressed associations as odds ratios (OR) with 95% CIs or their associated *p*-values. The interobserver agreement was calculated using Cohen's Kappa statistics or intraclass correlation efficiency assessed by observers A and B. The agreement between CDU and hrMRI for identifying features of EVAD was detected by Cohen's Kappa. Multiple collinearities of variables were diagnosed by tolerance <0.1 or variance inflation factor >10. Statistical significance was assumed if *p* < 0.05.

## Results

A total of 96 consecutive patients with unilateral EVAD identified by CDU and confirmed by hrMRI were enrolled. The median age of the study population was 38 years [(IQR) 33–48], and 47.9% of the population was men. Forty-one (42.7%) patients presented with symptoms of ischemic events (including forty patients with stroke and one with TIA), while fifty-five (57.3%) had local symptoms or signs only. Seven cases were identified with pulmonary infection and 4 cases with urinary tract infection within 1 week preceding the onset of symptoms caused by EVAD. In the ischemia group, five patients presented with stroke after severe trauma, and the interval between trauma and onset of stroke symptoms was 5 (±3) h, while the median time between trauma and symptoms in 17 patients with minor trauma (classified as spontaneous) before the ischemic events was 10 [5,42] h.

No significant differences were observed between patients with cerebral ischemia and those without in age, BMI, biochemical indexes, distribution of etiology, and the time intervals from symptom onset to CDU and hrMRI. However, a higher proportion of men and previous infections were noted in the ischemia group (69.5 vs. 34.5%; *p* = 0.002, and 19.5 vs. 5.5%; *p* = 0.070, respectively). Detailed patient demographics are listed in Table 1.

**Table 1:** Baseline characteristics of patients with EVAD-related ischemia and patients without ischemia.

	**Patients with ischemia (*****n** =* **41)**	**Patients without ischemia (*****n** =* **55)**	***P*** **-Value**
Age (mean ± SD) (year)	39.0 (32.5, 50.0)	38.0 (33.0, 46.0)	0.583
Age group (*n*) (%)			0.907
21–30	8 (19.5)	10 (18.2)	
31–40	14 (34.1)	24 (43.6)	
41–50	10 (24.4)	12 (21.8)	
51–60	7 (17.1)	7 (12.7)	
61–69	2 (4.9)	2 (3.6)	
Male (*n*) (%)	27 (65.9)	19 (34.5)	0.002
BMI (mean ± SD) (kg/m^2^)	22.3 ± 2.5	22.1 ± 2.1	0.681
Hypertension	10 (24.4)	7 (12.7)	0.139
Diabetes mellitus	3 (7.3)	2 (3.6)	0.735
Hyperlipidemia	4 (9.8)	10 (18.2)	0.247
Current smoking	6 (14.6)	2 (3.6)	0.120
Alcohol	4 (9.8)	1 (1.8)	0.205
Atrial fibfillation (*n*) (%)	0	0	–
Myocardial infarction (*n*) (%)	0	0	–
Biochemical index		
Total cholesterol (median) [IQR] (mmol/L)	3.8 (3.1, 4.4)	4.0 (3.4, 4.9)	0.216
Triglycerides (median) [IQR] (mmol/L)	1.4 (0.9, 1.8)	1.1 (0.7, 1.8)	0.117
HDL-C (mean ± SD) (mmol/L)	1.1 (0.9, 1.4)	1.2 (1.0, 1.5)	0.188
LDL-C (median) [IQR] (mmol/L)	2.4 ± 0.8	2.6 ± 0.7	0.219
hs-CRP (median) [IQR] (mg/L)	1.8 (0.7, 5.0)	1.6 (0.7, 3.2)	0.643
Previous infection (*n*) (%)	8 (19.5)	3 (5.5)	0.070
Urinary tract infection (*n*)	4	0	
Pulmonary infection (*n*)	4	3	
Etiology (*n*) (%)			0.419
Spontaneous	36 (87.8)	52 (94.5)	
Traumatic	5 (12.2)	3 (5.5)	
Time from onset to CDU (median) [IQR] (h)	5.0 (3.0, 8.5)	7.0 (3.0, 12)	0.293
Time group (*n*) (%)			0.510
≤ 6 h	25 (61.0)	27 (49.1)	
6–24 h	15 (36.6)	26 (47.3)	
25–48 h	1 (2.4)	2 (3.6)	
Time from onset to hrMRI (median) [IQR] (h)	6.0 (3.8, 11.0)	7.0 (4.5, 13)	0.250
Time group (*n*) (%)			0.727
≤ 6 h	22 (53.7)	25 (45.5)	
6–24 h	16 (39.0)	25(45.5)	
25–48 h	3 (7.3)	5 (9.1)	
Initial NIHSS (median) [IQR]	4 (2.0, 7.0)	0 (0.0, 0.0)	<0.001*
Initial mRS (median) [IQR]	4 (2.0, 4.0)	1 (1.0, 1.0)	<0.001*

Data are n (%) unless otherwise specified. BMI, body mass index; CDU, color duplex ultrasonography; HDL-C, high-density lipoprotein cholesterol; hrMRI, high-resolution magnetic resonance imaging; hs-CRP, high-sensitive C-reactive protein; LDL-C, low-density lipoprotein cholesterol; mRS, modified Rankin Scale; NIHSS, National Institutes of Health Stroke Scale; TIA, transient ischemic attack. *Significant for α= 0.05.

### Imaging characteristics of CDU and hrMRI

**Figure 1:**
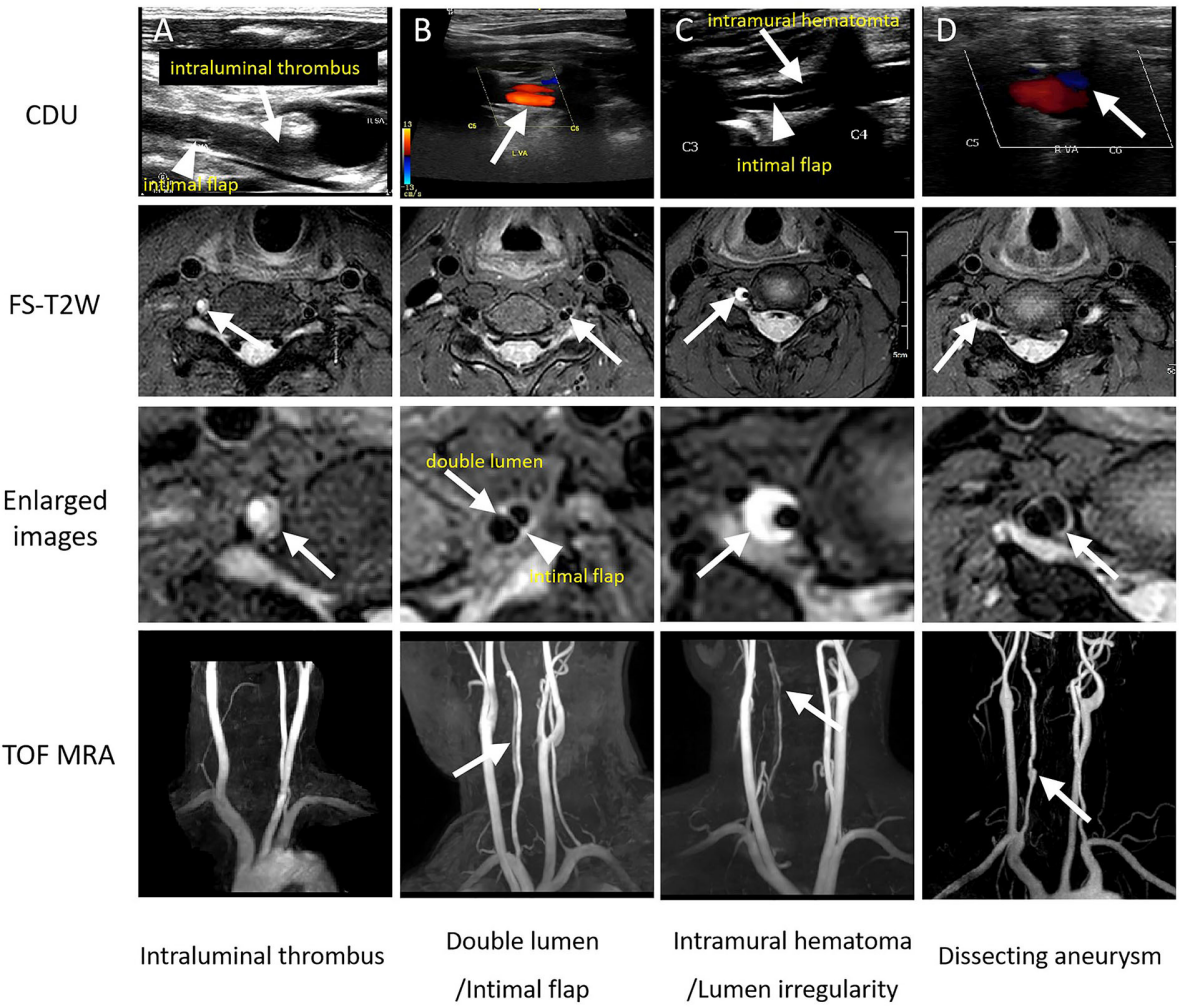
Characteristics of the extracranial vertebral artery dissection on color duplex ultrasonography (CDU) and its corresponding, pathognomonic high-resolution magnetic resonance imaging signs. The **(A–D)** four columns represent four patients (Patients # 1, 2, 3, and 4), respectively, and each column from top to bottom is CDU, fat-suppressed T2-weighted (FS-T2W), magnified view of FS-T2W, and 3D time-of-flight magnetic resonance angiography (TOF) imaging.

**Figure 2:**
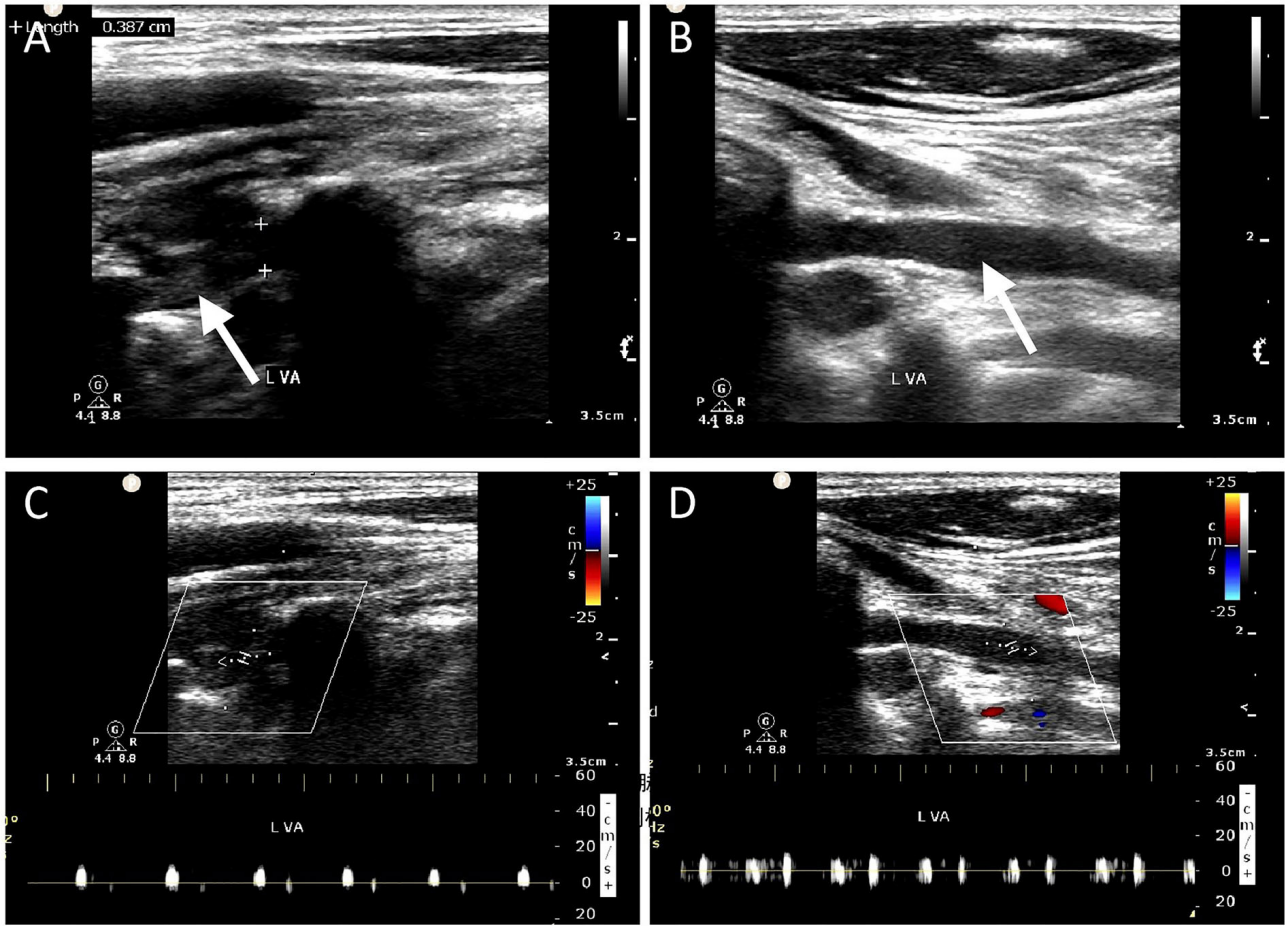
Ultrasonographic imaging for one case (Patient # 5) with left vertebral artery dissection. The 40-year-old man who had a history of falling down from a freight car 1 day ago presented with sudden syncope on admission. **(A)** Gray scale ultrasound examination reveals heterogeneous echo in the lumen, indicating intraluminal thrombus formation, which occupies the entire lumen of the V2 segment. **(B)** A homogenous echo in the V1 segment is suggestive of intramural hematoma. **(C,D)** No blood flow signal was detected, suggesting occlusion of the left VA.

Ultrasonographic characteristics of the two groups are summarized in Table 2. Images of four representative patients (Patients # 1, 2, 3, and 4) are shown in Figure 1. There was no significant difference in the external diameter of VA between patients with and without ischemia. However, the distribution of lesion location between the two groups was significantly different. Figure 1A (Patient #1) shows an intraluminal thrombus *in situ* identified by CDU forming directly at the intimal tear in the V1 segment, while Figure 2 (Patient # 5) depicts a distal thrombus forming downstream from the intramural hematoma. The detailed distribution of thrombus location is also shown in Table 2. Overall, intraluminal thrombus was observed mainly located at the distal part of lesions in the group of patients with acute ischemia.

**Table 2:** Imaging features on CDU and hrMRI of EVAD patients with and without ischemia.

**EVAD characteristics**	**Total** ***N** =* **96**	**Patients with ischemia (*****n** =* **41)**	**Patients without ischemia (*****n** =* **55)**	***P*** **-Value**
CDU
Intimal flap (*n*) (%)	92 (95.8)	41 (100.0)	51 (92.7)	0.133
Intramural hematoma (*n*) (%)	81 (84.4)	33 (80.5)	48 (87.3)	0.365
Double lumen (*n*) (%)	2 (2.1)	0 (0)	2 (3.6)	0.505
Intraluminal thrombus (*n*) (%)	18 (18.8)	15 (36.6)	3 (5.5)	<0.001*
Thrombus *in situ*		4 (9.8)	2(3.6)	
Thrombus at distal part of lesions		11 (26.8)	1 (1.8)	
Dissecting aneurysm (*n*) (%)	2 (2.1)	1 (2.4)	1 (1.8)	1.000
Lumen irregularity (*n*) (%)	62 (64.6)	26 (63.4)	36 (65.5)	0.836
Stenosis degree (*n*) (%)				0.001*
<50%	41 (42.7)	14 (34.1)	27 (49.1)	
50–69%	21 (21.9)	4 (9.8)	17 (30.9)	
70–99%	13 (13.5)	7 (17.1)	6 (10.9)	
100%	21 (21.9)	16 (39.0)	5 (9.1)	
Dissection site (*n*) (%)				0.008*
V1	8 (8.3)	7 (17.1)	1 (1.8)	
V2	41 (42.7)	16 (39.0)	25 (45.5)	
Distal V1-V2	30 (31.3)	8 (19.5)	22 (40.0)	
V1-V2	17 (17.7)	10 (24.4)	7 (12.7)	
Outer diameter (mean ± SD) (mm)	4.7 ± 0.9	4.8 ± 1.0	4.7 ± 0.9	0.764
Right side	53 (55.2)	22 (53.7)	31 (56.4)	0.792
hrMRI
Intimal flap (*n*) (%)	90 (93.8)	38 (92.7)	52 (94.5)	1.000
Intramural hematoma (*n*) (%)	81 (84.4)	32 (78.0)	49 (89.1)	0.141
Double lumen (*n*) (%)	2 (2.3)	0(0)	2 (3.6)	0.505
Intraluminal thrombus (*n*) (%)	17 (17.7)	14 (34.1)	3 (5.5)	<0.001*
Dissecting aneurysm (*n*) (%)	3 (3.5)	1 (2.4)	2 (3.6)	1.000
Lumen irregularity (*n*) (%)	63 (65.6)	26 (63.4)	37 (67.3)	0.694
Heterogeneous signal of IMH (*n*) (%)	38 (39.6)	20 (48.8)	18 (32.7)	0.112
Enhancement of IMH (*n*) (%)	22 (22.9)	12 (29.3)	10 (18.2)	0.201
Stenosis degree (*n*) (%)				0.003*
<50%	38 (38.4)	13 (31.7)	25 (45.5)	
50–69%	23 (24.0)	5 (12.2)	18(32.7)	
70–99%	16 (16.7)	9 (22.0)	7 (12.7)	
100%	19 (30.2)	14 (34.1)	5 (9.1)	
Lesion length (mean ± SD) (mm)	71.9 ± 36.6	81.5 ± 41.7	64.7 ± 30.8	0.025*
Outer diameter (mean ± SD) (mm)	4.9 ± 1.0	4.9 ± 1.1	4.9 ± 0.9	0.643
Right side (*n*) (%)	53 (55.2)	22 (53.7)	31 (56.4)	0.792

Data are n (%) unless otherwise specified. CDU, color duplex ultrasonography; hrMRI, high-resolution magnetic resonance imaging; IMH, intramural hematoma. *Significant for α= 0.05.

**Figure 3:**
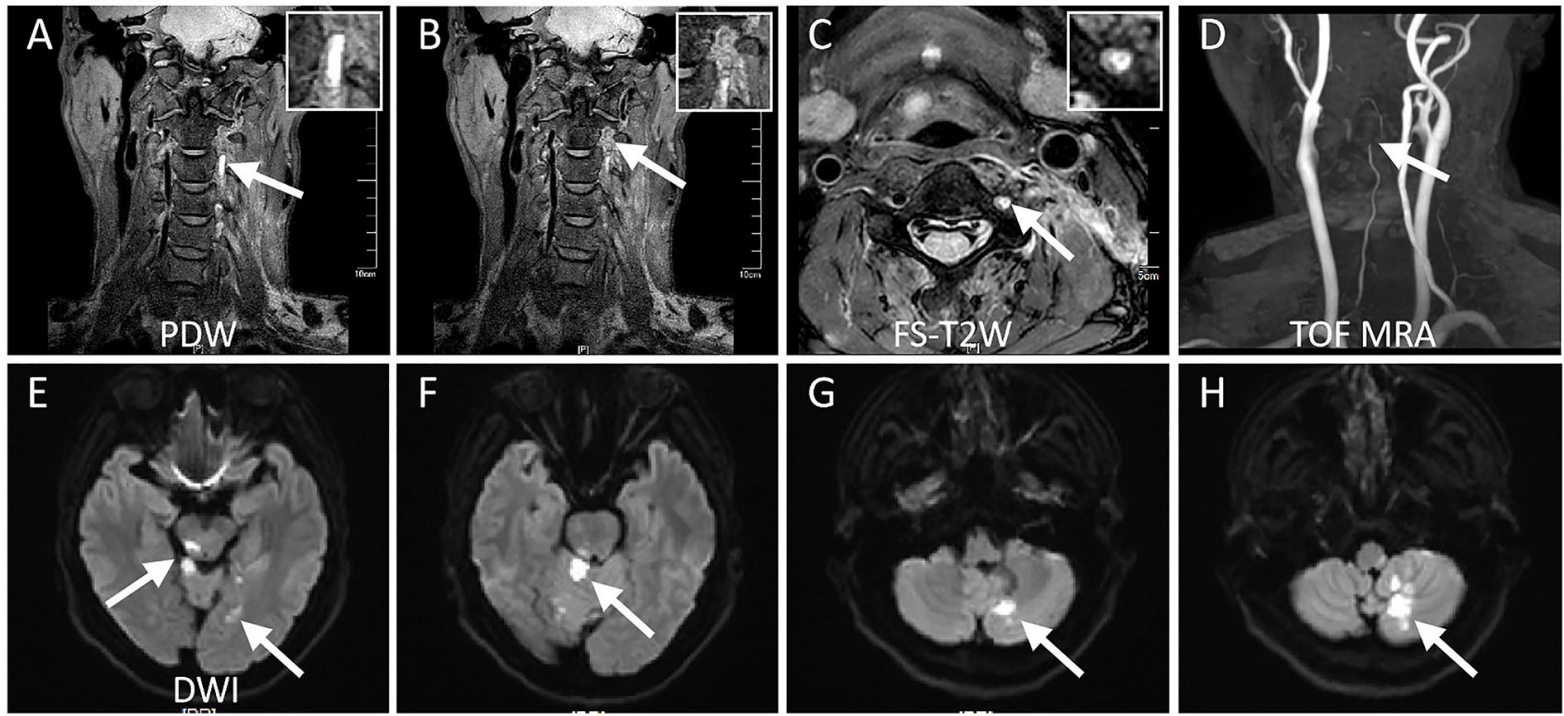
Magnetic resonance imaging studies for the same case (Patient # 5). **(A)** 3D proton density-weighted volumetric isotropic tse acquisition (PDW) shows the homogeneous hyperintensity within the lumen suggestive of intramural hematoma. **(B)** PDW demonstrates intraluminal thrombus formation with heterogeneous signals downstream of intramural hematoma. **(C)** Fat-suppressed T_2_-weighted axial sequence clearly shows the dilated lumen of the left VA fulfilling with heterogeneous hyperintensity, which symbolizes the formation of intraluminal thrombus as a donut sign, while a normal flow void is evident on the right VA. **(D)** 3D time of flight magnetic resonance angiography showing the lumen of left VA tapers gradually ending in total occlusion, which is indicative of a dissection. **(E–H)** Diffusion-weighted imaging demonstrates multiple, scattered, acute infarcts in posterior circulation territory suggestive of artery-to-artery embolization.

Details of the hrMRI features of the two groups are described in Table 2. Intramural hematoma appeared as a crescent-shape hyperintense on axial FS-T2W images, spiraling along the artery on 3D PDW images, resulting in varying degrees of concentric or eccentric stenosis. Intraluminal thrombus was identified as a typical donut sign on the axial FS-T2W images in Figure 3 (Patient # 5). Isolated or multiple acute infarctions in the posterior circulation territory were found in DWI images in all patients with ischemia, with a median pc-ASPECTS of 8 [7,9], while no acute infarcts were discovered in the non-ischemia group.

### The agreement between CDU and hrMRI findings

The agreement between CDU and hrMRI was almost perfect for the presence of double lumen (κ = 1.00); excellent for intraluminal thrombus (κ = 0.83), lumen irregularity (κ = 0.84), intramural hematoma (κ = 0.84), and dissecting aneurysm (κ = 0.80); and moderate for intimal flap (κ = 0.58). In addition, the agreement between CDU and hrMRI in evaluating luminal stenosis was excellent (κ = 0.85) (Table 3).

**Table 3:** The agreement between CDU and hrMRI findings.

	**CDU**	**hrMRI**	**Kappa**	**95%CI**	***P*** **-Value**
Intimal flap	92 (95.8)	90 (93.8)	0.58	0.21–0.95	<0.001
Intramural hematoma	81 (84.4)	81 (84.4)	0.84	0.69–0.99	<0.001
Double lumen	2 (2.1)	2 (2.3)	1.00	–	<0.001
Intraluminal thrombus	18 (18.8)	17 (17.7)	0.83	0.68–0.97	<0.001
Dissecting aneurysm	2 (2.1)	3 (3.5)	0.80	0.40–1.00	<0.001
Lumen irregularity	62 (64.6)	63 (65.6)	0.84	0.73–0.95	<0.001
Stenosis degree			0.85	0.77–0.94	<0.001
<50%	41 (42.7)	38 (38.4)			
50–69%	21 (21.9)	23 (24.0)			
70–99%	13 (13.5)	16 (16.7)			
100%	21 (21.9)	19 (30.2)			

*CDU, color duplex ultrasonography; hrMRI, high-resolution magnetic resonance imaging; IMH, intramural hematoma*.

### Association of imaging features and patient clinical presentations

In univariate regression analysis, intramural hematoma, dissecting aneurysm, and lumen irregularity in CDU were not significantly associated with stroke (Table 4). However, the presence of intraluminal thrombus and occlusion on CDU significantly contributed to ischemia. Additionally, lesions located at the V1 segment were associated with an increased risk of cerebral ischemia than those at the V2 or distal V1 extending to the V2 segment. In the multivariable logistic regression analysis, only intraluminal thrombus remained significant with the occurrence of ischemic events (*p* < 0.05), with an OR of 10.00 (95% CI, 2.66–37.66, *p* = 0.001).

**Table 4:** Characteristics of EVAD on CDU and hrMRI correlated to ischemic events.

**EVAD characteristics**	**Univariate**		**Multivariate**	
	**OR (95% CI)**	***P*** **-Value**	**OR** **(95% CI)**	***P*** **-Value**
CDU		
Intramural hematoma	0.60 (0.20–1.82)	0.368		
Intraluminal thrombus	10.00 (2.66–37.66)	0.001*	10.00 (2.66–37.66)	0.001*
Dissecting aneurysm	1.35 (0.08–22.24)	0.834		
Lumen irregularity	0.92 (0.39–2.13)	0.836		
Stenosis degree		
<50%	Reference	0.003*		
50–69%	0.45 (0.13–1.61)	0.221		
70–100%	2.25 (0.63–7.99)	0.210		
100%	6.17 (1.87–20.36)	0.003		
Dissection site		
V1	Reference	0.025*		
V2	0.09 (0.01–0.82)	0.032		
Distal V1–V2	0.05 (0.01–0.49)	0.010		
V1–V2	0.20 (0.02–2.05)	0.177		
Outer diameter	1.07 (0.69–1.68)	0.761		
Right side	1.12 (0.50–2.52)	0.792		
hrMRI		
Intimal flap	0.73 (0.14–3.82)	0.710		
Intramural hematoma	0.44 (0.14–1.34)	0.147		
Intraluminal thrombus	8.99 (2.38–34.01)	0.001*	8.99 (2.38–34.01)	0.001*
Dissecting aneurysm	0.66 (0.06–7.57)	0.740		
Lumen irregularity	0.84 (0.36–1.97)	0.694		
Heterogeneous signal of IMH	1.96 (0.85–4.50)	0.114		
Enhancement of IMH	1.86 (0.71–4.86)	0.204		
Stenosis degree		
<50%	Reference	0.006*		
50–69%	0.53 (0.16–1.77)	0.304		
70–99%	2.47 (0.75–8.16)	0.137		
100%	5.39 (1.59–18.26)	0.007		
Lesion length	1.01 (1.00–1.03)	0.028*		
Ourter diameter	0.90 (0.59–1.38)	0.639		
Right side	1.12 (0.50–2.52)	0.792		

CDU, color duplex ultrasonography; hrMRI, high-resolution magnetic resonance imaging; IMH, intramural hematoma. *Significant for α= 0.05.

As for hrMRI and MRA, the presence of intimal flap, intramural hematoma, double lumen, dissecting aneurysm, lumen irregularity, enhancement of intramural hematoma, or enlargement of outer contour were not significantly associated with ischemia (Table 3). However, intraluminal thrombus and occlusion were more frequent in patients with ischemia than in patients without, and the lesion length was longer in the ischemia group. In multivariable logistic regression analysis, only the presence of intraluminal thrombus was independently associated with acute ischemic events with an OR of 8.99 (95% CI, 2.38–34.01, *p* = 0.001).

We adjusted for other independent variables, including hypertension, and cigarette smoking, male gender, infections during the last week, and the presence of intraluminal thrombus on CDU and hrMRI were independently associated with acute ischemic events (Table 5).

**Table 5:** Clinical predictors and characteristics of on CDU and hrMRI images in patients with EVAD correlated to ischemic events.

**EVAD characteristics**	**Univariate**		**Multivariate**	
	**OR (95% CI)**	***P*** **-Value**	**OR (95% CI)**	***P*** **-Value**
Male	3.65 (1.56–8.57)	0.003	4.11 (1.54–10.92)	0.005
Hypertention	2.21 (0.76–6.43)	0.144		
Current smoking	4.54 (0.87–23.80)	0.073		
Previous infection	4.20 (1.04–16.99)	0.044	5.50 (1.13–26.87)	0.035
CDU		
Intraluminal thrombus	10.00 (2.66–37.66)	0.001*	7.38 (1.85–29.54)	0.005
Stenosis degree		
<50%	Reference	0.003*		
50–69%	0.45 (0.13–1.61)	0.221		
70–100%	2.25 (0.63–7.99)	0.210		
100%	6.17 (1.87–20.36)	0.003		
Dissection site		
V1	Reference	0.025*		
V2	0.09 (0.01–0.82)	0.032		
Distal V1–V2	0.05 (0.01–0.49)	0.010		
V1–V2	0.20 (0.02–2.05)	0.177		
Male	3.65 (1.56–8.57)	0.003	3.71 (1.40–9.84)	0.009
Hypertension	2.21 (0.76–6.43)	0.144		
Current smoking	4.54 (0.87–23.80)	0.073		
Previous infection	4.20 (1.04–16.99)	0.044	6.28 (1.33–29.69)	0.020
hrMRI		
Intraluminal thrombus	8.99 (2.38–34.01)	0.001*	6.10 (1.51–24.52)	0.011*
Stenosis degree		
<50%	Reference	0.006*		
50–69%	0.53 (0.16–1.77)	0.304		
70–99%	2.47 (0.75–8.16)	0.137		
100%	5.39 (1.59–18.26)	0.007		
Lesion length	1.01 (1.00–1.03)	0.028*		

*CDU, color duplex ultrasonography; hrMRI, high-resolution magnetic resonance imaging; IMH, intramural hematoma*.

*Significant for α= 0.05.

### Treatment outcome and predictors of unfavorable outcomes

A good functional outcome (mRS score 0–2) was observed in 84.4% of all patients at 6 months. In the multivariable analysis, a higher baseline NIHSS score (OR, 2.36; 95% CI, 1.62–3.43, *p* < 0.001) was the only independent predictor of unfavorable outcomes (Supplementary Tables S6–S9).

## Discussion

In our study sample, the median age was under 40 years, and ischemic events occurred in 42.7% of the patients identified with unilateral EVAD, lower than 89.0% in another large sample (1). Previous trauma occurred equally frequently in both the ischemia and non-ischemia groups. In addition, we identified three risk factors for ischemic events: male gender, infections during the last week, and the presence of intraluminal thrombus on CDU and hrMRI, respectively.

Although the exact mechanism of EVAD is yet to be fully elucidated, it is one of the predominant causes of posterior circulation ischemic stroke in young adults (28–30). In the present study, the male gender was found to be an independent predictor of cerebral ischemia in patients with unilateral EVAD. The association of male sex with cerebral ischemia is consistent with an analysis of 186 consecutive patients with spontaneous vertebral artery dissection (27), which indicated that ischemic events were more common in men, older patients, and smokers. However, there was no difference in smoking or age distribution between EVAD patients with and without ischemia in our cohorts. Some studies have confirmed that primary ischemic damage includes the disruption of neural cell mitochondria, which are essential to maintaining cellular energy and regulating oxidative stress (31), whereas estrogens and progestins in female both enhance mitochondrial respiration and augment anti-oxidative processes in neurons (32). Unfortunately, many gaps remain in the knowledge base of how sex and circulating sex hormones impact cerebrovascular disease processes, including EVAD-induced cerebral ischemia, on account of various factors that contribute to variability in testicular function in men, ovarian function among women, and variation in hormone levels associated with the menstrual cycle.

The present study included 11.5% of all patients having infections during the week preceding the onset of symptoms, suggesting that infection may be a trigger factor in the pathogenesis of EVAD. This association has been similarly observed in a case–control study (33), which reported that a recent infection was found in 31.9% of patients with spontaneous cervical artery dissection; however, no relationship was noted between the occurrence of recent infection and the type of clinical manifestation (with or without an ischemic event). Nevertheless, in our analysis, previous infections were associated with an increased risk for ischemia incidence, showing that inflammation might also be involved in the pathophysiology of cerebral ischemic events due to EVAD. In general, blood markers of inflammation are often elevated in acute stroke (34), which might have made it difficult to find the exact mechanism between infections and cerebral ischemia. Notwithstanding, further large-scale studies are needed in the future to validate and expand our conclusions.

Both in CDU and hrMRI, we found a higher prevalence of intraluminal thrombus and lumen occlusion in the ischemia group of patients with unilateral EVAD. Multivariable analysis showed that the presence of intraluminal thrombus on CDU and hrMRI was an independent risk factor associated with the occurrence of acute ischemic events, respectively. Intraluminal thrombus formation is commonly the result of local damage to the inner lining of an artery and is initiated by the activation of platelets, which is a complicated process with several biological and hemodynamic factors participating (35). According to certain observations, substantial shear stress in the tear region may have promoted the initial platelet activity (36, 37). Morel et al. (38) have indicated that thromboembolism, rather than hemodynamic infarction, is the most frequent cause of stroke in cervical artery dissection. Pelz and colleagues found evidence for a hypercoagulable state in patients with spontaneous cervical artery dissection as indicated by a shortened activated partial thromboplastin time, which was associated with a trend of an increased leukocyte count at the same time (39). Cerebral ischemic events might be caused by the damaged vessel wall in conjunction with the activation of the coagulation system, leading to thrombus formation. In general, it is considered that the determinants of arterial to arterial embolization in patients with EVAD include thrombus formation and continuous blood flow (40). The large presence of intraluminal thrombosis in our cohorts may be due to the study's long duration, which included different periods in which the stroke approach, including treatment and cure measures for EVAD, largely changed, and a few patients were not treated with thrombolysis because of delayed hospitalization, which may change the prognosis of the clinical syndrome. It should be noted that whether patients identified with EVAD could benefit from antithrombotic prophylaxis also needs to be further investigated in future studies, while immediate stroke prevention for EVAD patients without ischemic stroke is necessary (41–43).

Wu et al. (24) have demonstrated that the presence of irregular surface and intraluminal thrombus on hrMRI in cervicocranial artery dissection was independently associated with the occurrence of acute ischemic stroke. McNally et al. (10) reported that stenosis, intramural hematoma, dissecting aneurysm, male sex, current smoking, and nondissection stroke sources significantly contribute to stroke in multivariable regression. However, although both the above studies were based on large sample size, cervical and cerebral artery dissections were analyzed as a whole rather than classifying intracranial or extracranial artery dissection and anterior or posterior circulation in detail, and there may be differences in the causes and pathological mechanism of vascular dissection at different sites concerning stroke (44). The Cervical Artery Dissections and Ischemic Stroke Patients (CADISP) study demonstrated that patients with occlusive cervical artery dissection and multiple cervical artery dissections were more likely to develop delayed stroke (9). Remarkably, in our study, although the ischemia group exhibited a higher prevalence of occlusion in both CDU and hrMRI, the significance got lost in multivariate analysis. One possible explanation may be that when occlusion occurs on one side of the VA, cerebral perfusion of the posterior circulation can be maintained within a relatively normal range by collateral circulation construction, including compensation from the contralateral VA and/or rapid opening of the posterior communicating artery (45).

As a commonly used technique, CDU is considered non-invasive, economical, and available at the bedside, making it the preferred choice for screening cervical artery dissection (46, 47). Lu et al. (48) argued that the ultrasonographic features of vertebral artery dissection are determined by two major factors: the site of dissection and the severity of stenosis caused by dissection. Nevertheless, one drawback of CDU is that the V2 segment is invisible within the transverse bony foramens, limiting observation of the entire VA lumen (23). Therefore, the length of lesions cannot be directly measured on CDU. Instead, the lesions are located by anatomical structure. More importantly, to improve inspection accuracy and prevent missed diagnosis of localized lesions without significant stenosis, careful observation during the CDU examination should be conducted along the course of the vertebral artery, for its structure is not always completely straight and due to its long length.

While EVAD is thought of as a single entity, the diagnosis is met by various imaging findings, including dissection flap, intramural hematoma, double lumen, irregular stenosis, intraluminal thrombus, or dissecting aneurysm (13, 24). In the present study, CDU and hrMRI demonstrated consistent results in the depiction of these characteristics, and the combination of CDU and hrMRI was successful in showing the morphological characteristics of EVAD, and ultrasound imaging also allowed for the detection and assessment of complete recanalization over time. Previous studies mainly concentrated on the degree of stenosis and the composition of dissections contributing to stroke (29, 49). In the current study, although prominently longer lesions were observed on hrMRI in the ischemia group, statistical significance could not be confirmed in multivariable regression analysis.

Overall, 84.4% of all the patients achieved a favorable outcome at 6 months. It is worth noting that, in the multivariable analysis, none of the initial characteristics of CDU or hrMRI has a determinant effect on functional outcome in our patient cohort. In contrast, a higher baseline NIHSS was the only independent predictor for poor functional outcomes at 6 months, which was consistent with prior findings observed from previous studies focusing on risk factors for post-stroke functional outcomes (50).

There are several limitations to our study. First, this was a single-center, retrospective observational study with a small sample size. Second, intraluminal thrombus length was not quantified for evaluation, and contralateral VA and intracranial collateral circulation were not assessed. Third, the current study did not evaluate the inflammatory markers or leukocyte-related mediators that may provide further mechanistic insights. Strengths include that we have found independent associations between male gender, previous infections, the presence of intraluminal thrombus, and stroke occurrence in patients with unilateral EVAD. It may be useful for individual prediction and intervention of ischemic stroke early in EVAD, and immediate cervical imaging is also warranted in those without primary signs of stroke to avoid delayed cerebral ischemia, recurrent EVAD, and death.

## Conclusion

Male sex, infections during the last week, and intraluminal thrombus due to dissection are associated with an increased risk of ischemic events in patients with unilateral EVAD.

## Data availability statement

The raw data supporting the conclusions of this article will be made available by the authors, without undue reservation.

## Ethics statement

The studies involving human participants were reviewed and approved by the Medical Ethics Committee of the First Affiliated Hospital of Soochow University approved this study [(2021) No. 241]. The patients/participants provided their written informed consent to participate in this study. Written informed consent was obtained from the individual(s) for the publication of any potentially identifiable images or data included in this article.

## Author contributions

YY: conception and design. YY, ZL, YD, and CH: data collection. YY, ZL, and JP: analysis and interpretation of data. YY and ZL: drafting the article. ZT and PH: revising it critically for important intellectual content. All authors contributed to the article and approved the submitted version.

## Funding

This study was supported by the People's Livelihood Science and Technology Project (research on application of key technologies) of Suzhou (No. SS202061), Technical Cooperation Project of Soochow University (No. H211064), and Cadre Health Care Research Project of Jiangsu Province (No. BJ20009).

## Conflict of interest

The authors declare that the research was conducted in the absence of any commercial or financial relationships that could be construed as a potential conflict of interest. The handling editor J-CB declared a shared affiliation with the author ZT at the time of review.

## Publisher's note

All claims expressed in this article are solely those of the authors and do not necessarily represent those of their affiliated organizations, or those of the publisher, the editors and the reviewers. Any product that may be evaluated in this article, or claim that may be made by its manufacturer, is not guaranteed or endorsed by the publisher.

## Abbreviations

CDU: Color duplex ultrasonography
CT: Computed tomography
DWI: Diffusion-weighted image
EVAD: Extracranial vertebral artery dissection
hrMRI: high-resolution magnetic resonance imaging
NIHSS: National Institutes of Health Stroke Scale
TIA: Transient ischemic attack
VA: Vertebral artery.
