# Supplementary material for: Predictors of ischemic events in patients with unilateral extracranial vertebral artery dissection: A single-center exploratory study
Source: Front Neurol. 2022 Jul 28;13:939001. doi: 10.3389/fneur.2022.939001 (PMC9366300; doi:10.3389/fneur.2022.939001)
Supplement: Supplementary file 1 [file Data_Sheet_1.DOCX]

**hrMRI examination and assessment**

The hrMRI imaging parameters: T1W and CE-T1W, repetition time/ echo time (TR/TE)= 1000/20 ms, field of view (FOV)= 100×100 mm^2^, and matrix size= 352×352; FS-T2W, TR/TE= 3000/40 ms, FOV= 100×100 mm^2^, and matrix size= 384×384; PDW, TR/TE= 2000/33 ms, FOV= 200×200 mm^2^, and matrix size= 480×480; TOF MRA, TR/TE= 15/3.45 ms, FOV= 220×220 mm^2^, and matrix size= 336×336.

Brain imaging parameters: T1W, TR/TE= 2000/20 ms, FOV= 230×230 mm^2^, and matrix size= 512×512; T2W, TR/TE= 3000/90 ms, FOV= 230×230 mm^2^, and matrix size= 864×864; DWI, TR/TE= 2780/98 ms, FOV= 230×230 mm^2^, and matrix size= 192×192.

**Interobserver Agreement**

For the intrareader agreement in the identification of the presence of intimal flap, intramural hematoma, double lumen, intraluminal thrombus, dissecting aneurysm, irregular lumen, and stenosis degree on CDU, the correlation coefficient (*κ*) values were 0.79 (0.50-1.00), 0.80 (0.64-0.97), 1.00, 0.91(0.81-1.00), 1.00, 0.92 (0.83-1.00), and 0.93 (0.87-1.00), respectively. For the intrareader agreement in the identification of the presence of intimal flap, intramural hematoma, double lumen, intraluminal thrombus, dissecting aneurysm, irregular lumen, and stenosis severity on hrMRI, the *κ* values were 0.84 (0.63-1.00), 0.81 (0.65-0.97), 1.00, 0.88 (0.77-0.99), 1.00, 0.97 (0.92-1.00), and 0.88 (0.80-0.97), respectively. The interobserver intraclass correlation coefficient was 0.99 (0.99-1.00) for external diameter on CDU; 0.99 (0.92-1.00) for lesion length and 0.98 (0.95-0.99) for external diameter on hrMRI.
