# Supplementary material for: Predictors of ischemic events in patients with unilateral extracranial vertebral artery dissection: A single-center exploratory study
Source: Front Neurol. 2022 Jul 28;13:939001. doi: 10.3389/fneur.2022.939001 (PMC9366300; doi:10.3389/fneur.2022.939001)
Supplement: Supplementary file 2 [file Data_Sheet_2.docx]

**Table 6**  Baseline characteristics of EVAD patients with different outcomes at 6 months

|  | Unfavourable | Favourable | *P* Value |
| --- | --- | --- | --- |
|  | Yes ( n=15) | No ( n=81 ) |  |
| Age (median) [IQR] (year) | 35 (34, 54) | 38 (33, 47) | 0.650 |
| Age group (n) (%) |  |  | 0.927 |
| 21-30 | 3 (20.0) | 15 (18.5) |  |
| 31-40 | 5 (33.3) | 33 (40.7) |  |
| 41-50 | 3 (20.0) | 19 (23.5) |  |
| 51-60 | 3 (20.0) | 11 (13.6) |  |
| 61-69 | 1 (6.7) | 3 (3.7) |  |
| Male (n) (%) | 11 (73.3) | 35 (43.2) | 0.032* |
| BMI (mean ± SD) ( kg/m^2^) | 22.4 ± 2.0 | 22.1 ± 2.4 | 0.693 |
| Vascular risk factors (n) (%) |  |  |  |
| Hypertension | 4 (26.7) | 13 (16.0) | 0.534 |
| Diabetes mellitus | 2 (13.3) | 3 (3.7) | 0.363 |
| Hyperlipidemia | 3 (20.0) | 11 (13.6) | 0.803 |
| Current smoking | 2 (13.3) | 6 (7.4) | 0.799 |
| Alchhol | 2 (13.3) | 3 (3.7) | 0.363 |
| Previous infection (n) (%) | 1 (6.7) | 10 (12.3) | 0.847 |
| Biochemical index |  |  |  |
| Total cholesterol (median) [IQR] (mmol/L) | 3.8 (3.2, 4.4) | 3.9 (3.2, 4.6) | 0.467 |
| Triglycerides(mean ± SD) (mmol/L) | 1.4 (0.6, 1.8) | 1.1 (0.8, 1.7) | 0.988 |
| HDL-C (median) [IQR] (mmol/L) | 1.1 (0.9, 1.4) | 1.1 (0.9, 1.5) | 0.679 |
| LDL-C (mean ± SD) (mmol/L) | 2.5 ± 0.9 | 2.5 ± 0.7 | 0.922 |
| hs-CRP (median) [IQR] (mg/L) | 1.7 (0.7, 2.6) | 1.9 (0.8, 3.3) | 0.653 |
| Etiology (n) (%) |  |  | 0.799 |
| [Spontaneous](C:/Users/Lenovo/AppData/Local/youdao/dict/Application/8.9.6.0/resultui/html/index.html#/javascript:;) | 13 (86.7) | 75 (92.6) |  |
| Traumatic | 2 (13.3) | 6 (7.4) |  |
| Time from onset to CDU (median) [IQR] (hours) | 5.0 (3.0, 12.0) | 6.0 (3.0, 12.0) | 0.686 |
| Time group (n) (%) |  |  | 0.488 |
| ≤6 h | 10 (66.7) | 42 (51.9) |  |
| 6 ～24 h | 5 (33.3) | 36 (44.4) |  |
| 25～48 h | 0 (0) | 3 (3.7) |  |
| Time from onset to hrMRI (median) [IQR] (hours) | 5.5 (3.5, 13.0) | 7.0 (4.0, 12.5) | 0.582 |
| Time group (n) (%) |  |  | 0.648 |
| ≤6 h | 9 (60.0) | 38 (46.9) |  |
| 6 ～24 h | 5 (33.3) | 36(44.4) |  |
| 25～48 h | 1 (6.7) | 7 (8.6) |  |
| Initial NIHSS (median) [IQR] | 7 (5.0, 8.0) | 0 (0.0, 1.0) | <0.001* |
| Admission stroke severity (n) (%) |  |  | <0.001* |
| Mild (NIHSS 1-4) | 0 (0) | 57 (70.4) |  |
| Moderate (NIHSS 5-15) | 1 (6.7) | 17 (21.0) |  |
| Moderate to severe (NIHSS 16-20) | 14 (93.3) | 7 (8.6) |  |
| Severe (NIHSS 21-42) | 0 | 0 |  |
| Treatment (n) (%) |  |  | <0.001* |
| Antiplatelet | 5 (33.3) | 52 (64.2) |  |
| Anticoagulation | 3 (20.0) | 11 (13.6) |  |
| Antiplatelet + Anticoagulation | 1 (6.7) | 17 (21.0) |  |
| Intravenous thrombolysis | 6 (40.0) | 1 (1.2) |  |

Abbreviations: BMI, body mass index; CDU, color duplex ultrasonography; HDL-C, high-density lipoprotein cholesterol; hrMRI, high-resolution magnetic resonance imaging; hs-CRP, high-sensitive C-reactive protein; LDL-C, low-density lipoprotein cholesterol; mRS, modified Rankin Scale; NIHSS, National Institutes of Health Stroke Scale; TIA, transient ischemic attack. *Significant for α= 0.05.

**Table 7**  Initail CDU and hrMRI characteristics correlated to functional outcomes at 6 months

| EVAD characteristics | Total  N=96 | Unfavourable | Favourable | *P* Value |  |
| --- | --- | --- | --- | --- | --- |
|  |  | Yes ( n=15) | No ( n=81 ) |  |  |
| CDU | | | | |  |
| Intimal flap (n) (%) | | 92 (95.8) | 15 (100.0) | 77 (95.1) | 1.000 |
| Intramural hematoma (n) (%) | | 81 (84.4) | 12 (80.0) | 69 (85.2) | 0.904 |
| Double lumen (n) (%) | | 2 (2.1) | 0 (0) | 2 (2.5) | 1.000 |
| Intraluminal thrombus (n) (%) | 18 (18.8) | 6 (40.0) | 12(14.8) | 0.053* |  |
| Thrombus in situ |  | 2 (13.3) | 4 (4.9) |  |  |
| Thrombus at distal part of lesions |  | 4 (26.7) | 8 (9.9) |  |  |
| [Dissecting](C:/Users/Lenovo/AppData/Local/youdao/dict/Application/8.9.6.0/resultui/html/index.html#/javascript:;) [aneurysm](C:/Users/Lenovo/AppData/Local/youdao/dict/Application/8.9.6.0/resultui/html/index.html#/javascript:;) (n) (%) | 2 (2.1) | 1 (6.7) | 1 (1.2) | 0.289 |  |
| Lumen irregularity (n) (%) | | 62 (64.6) | 8 (53.3) | 54 (66.7) | 0.321 |
| Stenosis degree (n) (%) |  |  |  | 0.037* |  |
| < 50% | 41 (42.7) | 4 (26.7) | 37 (45.7) |  |  |
| 50⁓69% | 21 (21.9) | 1 (6.7) | 20 (24.7) |  |  |
| 70⁓99% | 13(40.7) | 3 (20.0) | 10 (12.3) |  |  |
| 100% | 21(21.9) | 7(46.7) | 14(17.3) |  |  |
| Dissection site (n) (%) |  |  |  | 0.021* |  |
| V1 | 8 (8.3) | 4 (26.7) | 4 (4.9) |  |  |
| V2 | 41 (42.7) | 4 (26.7) | 37 (45.7) |  |  |
| Distal V1-V2 | 30 (31.3) | 3(20.0) | 27 (33.3) |  |  |
| V1-V2 | 17 (17.7) | 4(26.7) | 13 (16.0) |  |  |
| Outer diameter (mean ± SD) (mm) | 4.7 ± 0.9 | 4.9 ± 1.1 | 4.7 ± 0.9 | 0.437 |  |
| Right side | 53 (55.2) | 5 (33.3) | 48 (59.3) | 0.064* |  |
| hrMRI | | | | |  |
| Intimal flap (n) (%) | 90 (93.8) | 13 (86.7) | 77 (95.1) | 0.217 |  |
| Intramural hematoma (n) (%) | 81 (84.4) | 12 (80.0) | 69 (85.2) | 0.904 |  |
| Double lumen (n) (%) | 2 (2.1) | 0(0) | 2 (2.5) | 1.000 |  |
| Intraluminal thrombus (n) (%) | 17 (17.7) | 6 (40.0) | 11 (13.6) | 0.036* |  |
| [Dissecting](C:/Users/Lenovo/AppData/Local/youdao/dict/Application/8.9.6.0/resultui/html/index.html#/javascript:;) [aneurysm](C:/Users/Lenovo/AppData/Local/youdao/dict/Application/8.9.6.0/resultui/html/index.html#/javascript:;) (n) (%) | 3 (3.1) | 1 (6.7) | 2 (2.5) | 0.403 |  |
| Lumen irregularity (n) (%) | 63 (65.6) | 10 (66.7) | 53 (65.4) | 0.926 |  |
| Heterogeneous signal of IMH (n) (%) | 38 (39.6) | 9 (60.0) | 29 (35.8) | 0.078 |  |
| Enhancement of IMH (n) (%) | 22 (22.9) | 6 (40.0) | 16 (19.8) | 0.168 |  |
| Stenosis degree (n) (%) |  |  |  | 0.053 |  |
| < 50% | 38 (39.6) | 4 (26.7) | 34(42.0) |  |  |
| 50⁓69% | 23 (24.0) | 1 (6.7) | 22 (27.2) |  |  |
| 70⁓99% | 16 (16.7) | 4 (26.7) | 12 (14.8) |  |  |
| 100% | 19 (19.8) | 6 (40.0) | 13 (16.0) |  |  |
| Lesion length (mean ± SD) (mm) | 71.9 ± 36.6 | 76.6 ± 44.3 | 71.0 ± 36.1 | 0.591 |  |
| Outer diameter (mean ± SD) (mm) | 4.9 ± 1.0 | 5.1± 1.3 | 4.9 ± 0.9 | 0.523 |  |
| Right side (n) (%) | | 53 (55.2) | 5 (33.3) | 48 (59.3) | 0.064* |

Abbreviations: CDU, color duplex ultrasonography; hrMRI, high-resolution magnetic resonance imaging; IMH, intramural hematoma.

*Significant for α= 0.05.

**Table 8** Inital CDU and hrMRI characteristics correlated to unfavourable outcomes

| EVAD characteristics | Univariate | | Multivariate | |  |
| --- | --- | --- | --- | --- | --- |
|  | OR ( 95% CI ) | *P* Value | OR ( 95% CI ) | *P* Value |  |
| CDU | | | | |  |
| Intramural hematoma | | 0.70 (0.17-2.84) | 0.613 |  |  |
| Intraluminal thrombus | 3.83 (1.15-12.74) | 0.028* |  |  |  |
| [Dissecting](C:/Users/Lenovo/AppData/Local/youdao/dict/Application/8.9.6.0/resultui/html/index.html#/javascript:;) [aneurysm](C:/Users/Lenovo/AppData/Local/youdao/dict/Application/8.9.6.0/resultui/html/index.html#/javascript:;) | 5.71 (0.34-96.77) | 0.227 |  |  |  |
| Lumen irregularity | | 0.57 (0.19-1.74) | 0.325 |  |  |
| Stenosis degree |  |  |  |  |  |
| < 50% | Reference | 0.064 |  |  |  |
| 50⁓69% | 0.46 (0.05-4.42) | 0.503 |  |  |  |
| 70⁓99% | 2.78 (0.53-14.48) | 0.226 |  |  |  |
| 100% | 4.63(1.17-18.27) | 0.029* |  |  |  |
| Dissection site |  |  |  |  |  |
| V1 | Reference | 0.046* | Reference | 0.013 |  |
| V2 | 0.11 (0.02-0.61) | 0.012* | 0.03 (0.004-0.30) | 0.002 |  |
| Distal V1-V2 | 0.11 (0.02-0.69) | 0.019* | 0.03 (0.003-0.33) | 0.004 |  |
| V1-V2 | 0.31(0.05-1.83) | 0.195 | 0.13 (0.02-1.06) | 0.056 |  |
| Outer diameter | 1.27 (0.70-2.30) | 0.434 |  |  |  |
| Right side | 2.91 (0.91-9.29) | 0.072 | 7.77 (1.53-39.56) | 0.014 |  |
| hrMRI | | | | |  |
| Intimal flap | 1.18 (0.13-10.66) | 0.882 |  |  |  |
| Intramural hematoma | 0.34 (0.06-2.04) | 0.236 |  |  |  |
| Intraluminal thrombus | 4.24 (1.26-14.27) | 0.020* | 4.24 (1.26-14.27) | 0.020 |  |
| [Dissecting](C:/Users/Lenovo/AppData/Local/youdao/dict/Application/8.9.6.0/resultui/html/index.html#/javascript:;) [aneurysm](C:/Users/Lenovo/AppData/Local/youdao/dict/Application/8.9.6.0/resultui/html/index.html#/javascript:;) | 2.82 (0.24-33.25) | 0.410 |  |  |  |
| Lumen irregularity | 1.06 (0.33-3.39) | 0.926 |  |  |  |
| Heterogeneous signal of IMH | 2.69 (0.87-8.31) | 0.086 |  |  |  |
| Enhancement of IMH | 2.71 (0.84-8.72) | 0.095 |  |  |  |
| Stenosis degree |  |  |  |  |  |
| < 50% | Reference | 0.089 |  |  |  |
| 50⁓69% | 0.39 (0.04-3.69) | 0.409 |  |  |  |
| 70⁓99% | 2.83 (0.61-13.14) | 0.183 |  |  |  |
| 100% | 3.92 (0.95-16.19) | 0.059 |  |  |  |
| Lesion length | 1.00 (0.99-1.02) | 0.587 |  |  |  |
| Ourter diameter | 1.20 (0.69-2.07) | 0.520 |  |  |  |
| Right side | 2.91 (0.91-9.29) | 0.072 |  |  |  |

Abbreviations: CDU, color duplex ultrasonography; hrMRI, high-resolution magnetic resonance imaging; IMH, intramural hematoma.

*Significant for α= 0.05.

**Table 9** Predictors of unfavorable outcomes in 96 patients with unilateral EVAD

|  | Univariate | | Multivariate | |
| --- | --- | --- | --- | --- |
|  | OR ( 95% CI ) | *P* Value | OR ( 95% CI ) | *P* Value |
| Male | 3.61 (1.06-12.32) | 0.040 |  |  |
| Initial NIHSS | 2.36 (1.62-3.43) | <0.001 | 2.36 (1.62-3.43) | <0.001 |
| Treatment |  |  |  |  |
| Antiplatelet | Reference | 0.003 |  |  |
| Anticoagulation | 2.84 (0.59-13.66) | 0.194 |  |  |
| Antiplatelet + Anticoagulation | 0.61 (0.07-5.61) | 0.664 |  |  |
| Intravenous thrombolysis | 62.40 (6.21-626.98) | <0.001 |  |  |
| CDU |  |  |  |  |
| Dissection site |  |  |  |  |
| V1 | Reference | 0.046 |  |  |
| V2 | 0.11 (0.02-0.61) | 0.012 |  |  |
| Distal V1-V2 | 0.11 (0.02-0.69) | 0.019 |  |  |
| V1-V2 | 0.31(0.05-1.83) | 0.195 |  |  |
| Right side | 2.91 (0.91-9.29) | 0.072 |  |  |
| hrMRI |  |  |  |  |
| Intraluminal thrombus | 6.30 (1.85-21.47) | 0.003 |  |  |

Abbreviations: CDU, color duplex ultrasonography; hrMRI, high-resolution magnetic resonance imaging; NIHSS, National Institutes of Health Stroke.
